# Identification of the pedunculopontine nucleus and surrounding white matter tracts on 7T diffusion tensor imaging, combined with histological validation

**DOI:** 10.1007/s00276-018-2120-3

**Published:** 2018-10-31

**Authors:** D. J. H. A. Henssen, D. Kuppens, F. J. A. Meijer, A. M. van Cappellen van Walsum, Y. Temel, E. Kurt

**Affiliations:** 1 Department of Neurosurgery, Unit of Functional Neurosurgery, Radboud University Medical Center, Nijmegen, The Netherlands; 2 Department of Anatomy, Radboud University Medical Center, Geert Grooteplein Noord 25, 6500 HB Nijmegen, The Netherlands; 3 Donders Institute for Brain, Cognition and Behavior, Radboud University Medical Center, Nijmegen, The Netherlands; 4 Department of Radiology and Nuclear Medicine, Radboud University Medical Center, Nijmegen, The Netherlands; 5 Department of Neurosurgery and Neuroscience, Maastricht University Medical Center, Maastricht, The Netherlands

**Keywords:** Pedunculopontine nucleus, Diffusion tensor imaging, Parkinson’s disease, Deep brain stimulation

## Abstract

**Background:**

The pedunculopontine nucleus (PPN) has been studied as a possible target for deep brain stimulation (DBS) for Parkinson’s disease (PD). However, identifying the PPN can be challenging as the PPN is poorly visualized on conventional or even high-resolution MR scans. From histological studies it is known that the PPN is surrounded by major white matter tracts, which could function as possible anatomical landmarks.

**Methods:**

This study aimed to localize the PPN using 7T magnetic resonance (MR) imaging and diffusion tensor imaging (DTI) of its white matter borders in one post-mortem brain. Histological validation of the same specimen was performed. The PPN was segmented in both spaces, after which the two masks were compared using the Dice Similarity Index (DSI). The DSI compared the similarity of two samples on an inter-individual level and validated the MR findings. The error in distance between the center of the two 3D segmentations was measured by use of the Euclidean distance.

**Results:**

The PPN can be found in between the superior cerebellar peduncle and the medial lemniscus on both the FA-maps of the DTI images and the histological sections. The histological transverse sections showed to be superior to recognize the PPN (DSI: 1.0). The DTI images have a DSI of 0.82. The overlap-masks of both spaces showed a DSI of 0.32, whereas the concatenation-masks of both spaces showed a remarkable overlap, a DSI of 0.94. Euclidean distance of the overlap- and concatenation-mask in the two spaces showed to be 1.29 mm and 1.59 mm, respectively.

**Conclusion:**

This study supports previous findings that the PPN can be identified using FA-maps of DTI images. For possible clinical application in DBS localization, in vivo validation of the findings of our study is needed.

## Introduction

The pedunculopontine nucleus (PPN) is a locomotor center processing sensory and behavioral information, presumably executing a function within the mechanisms of axial symptoms and postural instability [13]. This broad description of functions of PPN is based on its extensive connectivity [15]. The PPN itself can be found in the ventrolateral part of the caudal mesencephalic tegmentum at the junction of the pons and midbrain at the level of the inferior colliculus and is known to extend in a rostral direction towards the caudal pole of the red nucleus. The cytoarchitecture of the PPN divides the nucleus into two subnuclei: the subnucleus compactus (dorsolateral portion) and the subnucleus dissipatus (ventromedial portion) [22]. On a transverse section, the PPN is mainly bounded by multiple white matter tracts. Medially, the superior cerebellar peduncle (SCP) can be found, whereas the lateral and ventral borders are made up by the fibers of the medial lemniscus (ML) and the decussating SCP. The dorsal border is formed by the cuneiform nucleus and the ventral mesencephalic reticular formation. Because of its function within mechanisms of motor function, there has been an increase in interest in PPN as a target for deep brain stimulation to treat Parkinson’s disease related and dopaminergic treatment resistant gait and balance dysfunction [12].

Targeting the PPN based on MR imaging is complicated by the lack of distinction between the PPN and adjacent neural structures on T1- and T2-weighted Magnetic Resonance (MR)sequences [23]. Other MR techniques, such as diffusion-weighted MR imaging (DW-MRI) and diffusion tensor imaging (DTI), could be a valuable addition in the recognition of the PPN in clinical settings. The use of DTI to recognize the course of white matter tracts that are difficult to identify on structural MR scans has proved to be of great value, both in neurosurgical practice [28] as in other clinical settings [1, 17]. As the PPN is bordered by large white matter tracts, DTI could be a valid method to visualize the PPN in an indirect manner. Recently, Alho et al. reported an imaging method that can possibly provide patient-specific imaging markers to facilitate the targeting of the PPN, based on fractional anisotropy (FA) values in DTI [4]. The random motion of water molecules is restricted by the normal architecture of neuroglial tissue and fiber tracts, which is called anisotropy. The degree of anisotropy can be quantified by applying diffusion sensitizing gradients in at least six directions from which fractional anisotropy (FA) is calculated. It is known that fiber bundles restrict the movement of water molecules more than gray matter. The findings of Alho et al. indicated that the PPN could be delineated by a contrast in FA-values from the surrounding tissue, including the white matter fiber bundles (high values) and inferior colliculus (low values).

This study aims to identify the PPN by combining high-resolution post-mortem MRI with histological validation. The availability of a 7T-diffusion weighted magnetic resonance (DW-MR) imaging scanner provides the opportunity to assess the possible imaging markers, using DTI, for the PPN as discussed by Alho et al. with a higher resolution and improved contrast-to-noise ratio [3]. Furthermore, as the study from Alho et al. showed that identification of the PPN is possible using the white matter borders [3], a myelin staining was carried out to validate the high-resolution DTI findings of our study.

## Materials and methods

### Sample acquisition

A post-mortem brain of an 87 year old specimen was acquired via the body donor program at the Department of Anatomy of the Radboud University Medical Centre, Nijmegen, The Netherlands. Cause of death showed to be pneumonia and the subject had no prior neurological or psychiatric diseases. As determined by two neuroanatomists, gross morphology of the brain, as well as the serial sections, showed no pathological changes. 10 h after death, the body was perfused with formalin via the femoral artery to allow internal fixation of the tissue. After approximately 24 h, the brain was extracted from the skull and stored in 7.7% formalin for 16 months. Frontal, temporal, parietal, and occipital lobes were removed to fit the specimen in the smaller coil of the MR-scanner for high signal reception. Prior to scanning, the specimen was immersed in a phosphate-buffered saline solution for 72 h to remove formalin from the tissue, as formalin is known to decrease the T2 relaxation rate of tissue [25]. Furthermore, fixed tissues are known to suffer from reduced apparent diffusion coefficients (ADC) [7, 26, 27], requiring higher b values to obtain similar diffusion contrast as for in vivo. Studies also reported a reduction of the fractional anisotropy (FA) values in post-mortem white matter [6, 24]. The relatively short postmortem interval (period of time between death and fixation) that was employed in this specimen limited the reduction in ADC and FA [6]. Further, diffusivity measures have been suggested to remain stable up to 3 years after fixation [9].

### MR imaging

All imaging was performed on a Siemens MAGNETOM 7T MRI scanner. A 28-channel knee coil (Siemens, Erlangen, Germany) was used for data acquisition. Structural images were acquired with a true fast imaging steady-state free precession (TRUFI) sequence, at 0.4 mm isotropic resolution and 0.5 mm slice thickness [18]. Because T1 and T2 estimates are required for the analysis of the DW-SSFP data, true inversion recovery (TIR) and turbo spin echo (TSE) adopted for 7T were included in the scanning protocol [10]. DTI images were acquired at 1.0 mm isotropic resolution with a 1.4 mm slice thickness and an effective b value of 5175s/mm2 in 49 directions (two averages). As general reduction of the ADC and FA occurs in fixated tissue, a diffusion-weighted steady-state free precession (DW-SSFP) sequence was implemented in the scanning protocol [6, 24]. The presented protocol resulted in a total scanning time of approximately 32 h. The applied scanning protocol has been previously described in more detail by Mollink et al. [19]. Images were viewed using the FMRIB (functional magnetic resonance imaging of the brain) software library (FSL) (FMRIB Software Library, FSL (c) 2012, The University of Oxford). Table 1 provides a comprehensive overview of the applied acquisition characteristics.

**Table 1:** Characteristics of the applied diffusion-weighted, MRI protocol

Diffusion-weighted steady-state free precession (DW-SSFP)	
*T*_E_	21 ms
*T*_R_	30 ms
*α*	30°
Number of directions	49
Number of averages	2
Number of *q* = 10/mm (*b* = 0 equivalent)	8
Matrix size	176 × 120 × 180 mm^3^
Voxel size	1.0 × 1.0 × 1.0 mm^3^
Bandwidth	80 Hz/pixel
*b* value (equivalent)	~ 5175 s/mm^2^
*q* value	300/mm

True fast imaging with steady-state free precession (TRUFI, anatomical)	
*T*_E_	3.79 ms
*T*_R_	7.58 ms
*α*	35°
Matrix size	416 × 256 × 512 mm
Voxel size	0.4 × 0.4 × 0.5 mm
Bandwidth	296 Hz/pixel

${T_E}$ Echo time, represents the time from the center of the RF-pulse to the center of the echo; *T*_*R*_ repetition time, the length of time between corresponding consecutive points on a repeating series of pulses and echoes; *α* flip angle, amount of rotation the magnetization experiences during application of a radiofrequency (RF) pulse; *b value* measures the degree of diffusion weighting applied; *q value b* value in DW-SSFP imaging, proportional to the diffusion-encoding

### Ethical statement

This study was carried out in accordance with the recommendations of the CMO (Commissie Mensgebonden Onderzoek) region Arnhem-Nijmegen, Netherlands. Also, the protocol was approved by the CMO region Arnhem-Nijmegen, Netherlands. The specimen was acquired via the body donor program at the department of anatomy of the Radboud University Medical Centre, Nijmegen, Netherlands. All body donors in this program signed a written informed consent during lifetime permitting the use of their body and parts for scientific research and educational purposes.

### Histological sectioning and staining

**Fig. 1:**
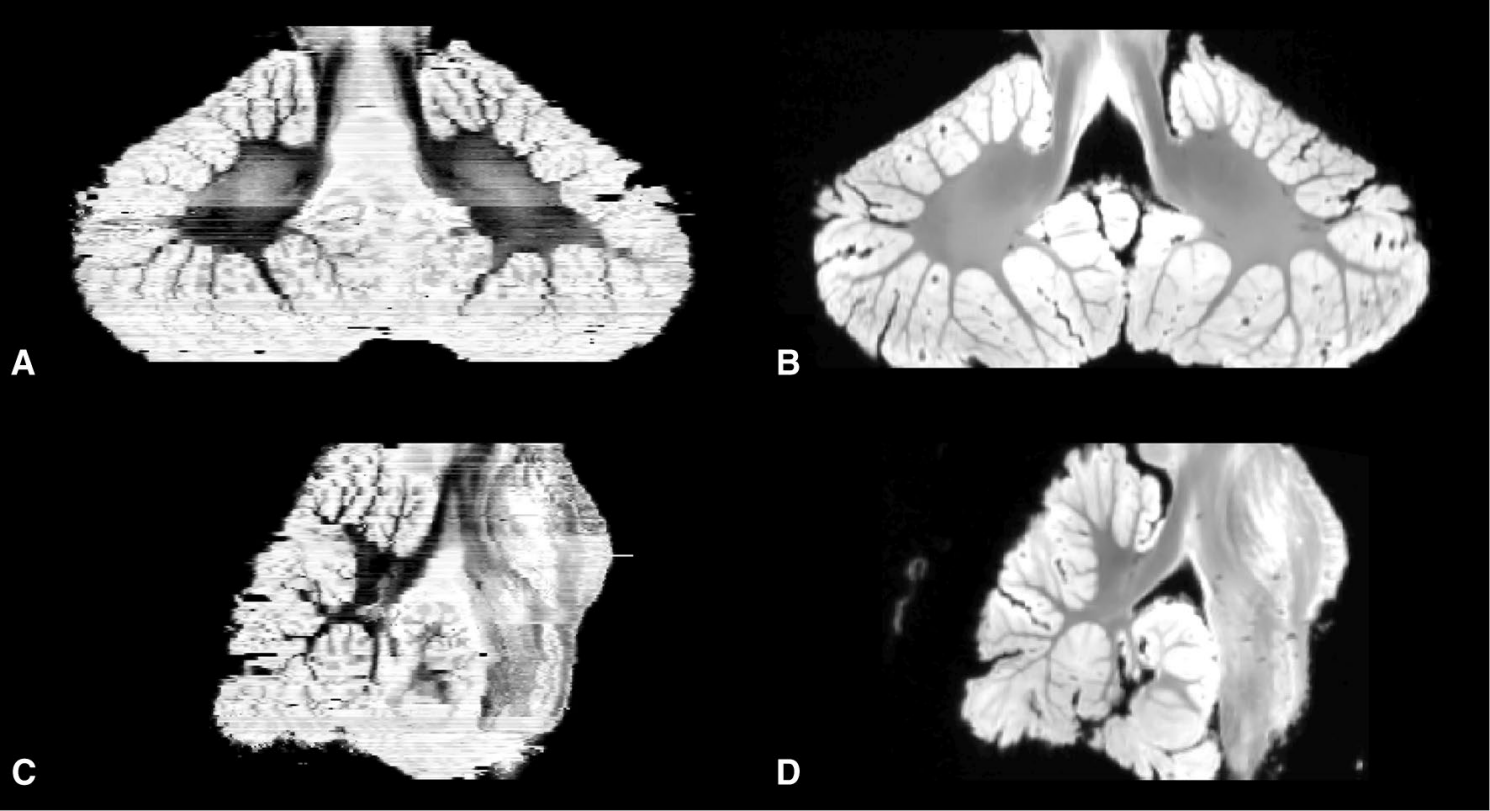
Reconstruction of the histological sections from the cerebellum and brainstem. Following affine registration, a non-linear registration approach was applied and represented in a coronal (**a**) and sagittal view (**c**). Alignment of internal structures significantly improved after this step and was most pronounced in white matter. Corresponding MRI slices for **a** and **c** are depicted in **b** and **d**, respectively

The histological collection as described by Mollink et al. was used [19]. The scanned specimen was serially sectioned using a heavy-duty LKB 2260 Macrotome (LKB Instruments, Bromma, Sweden) at a thickness of 10 µm with an interslice distance of 200 µm. Myelin staining of the sections was performed with the modified Heidenhain-Woelke stain [5]. All sections were digitized and registered to create a histological volume using a 2D affine transformation based on at least six landmarks. With use of a 3D affine registration with FLIRT [14], the histological volume was registered with both the structural and DTI images. A 2D affine landmark-based registration was used to perform repetitive alignments of the histological images to their corresponding MRI slices. This process was repeated 20 times and resulted in a very accurate histological three-dimensional reconstruction (Fig. 1).

### Segmentation, dice similarity indices and Euclidean distance

**Fig. 2:**
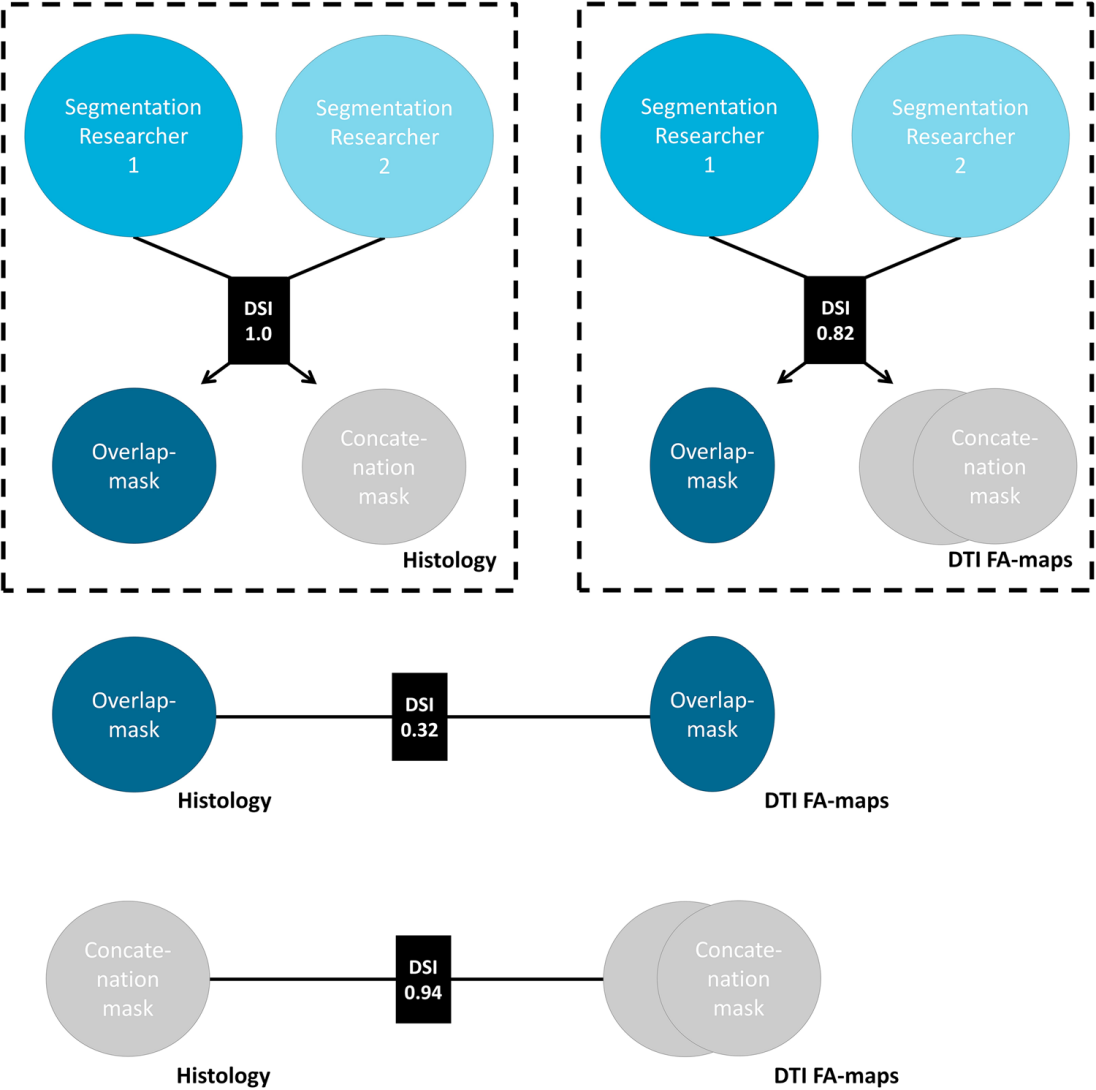
Work-flow of segmentation and dice similarity indices. Overlap-masks compare the common ground of different segmentations; Concatenation-masks combine the grounds of the different segmentations. *DSI* Dice similarity indices; *DTI FA-maps* diffusion tensor imaging fractional anisotropy map

The location of the PPN in the histological sections was identified by a third investigator and neuroanatomist (AMvCvW) using neuroanatomical landmarks that were derived from the literature [20–22]. Mesencephalic landmarks that were used were (1) the inferior colliculus, (2) the SCP and (3) the ML. Subsequently, two investigators (DK and DH) independently performed manual segmentation of the PPN in the DTI images and in the histological sections. The PPN was segmented in the transverse planes of the DTI images first, after which the sagittal and coronal planes were used to adjust the segmentation. FA-values of the segmented areas were measured and varied between 0 and 1.0, 0 being isotropic and 1.0 being anisotropic. Subsequently, the segmented images were converted into an overlap-mask and a concatenation-mask. The overlap-mask measured the common ground of both masks, whereas the concatenation-mask covers the sum of the areas of both masks. The Dice Similarity Index (DSI) (Eq. 1) was calculated to measure the overlap between the segmentations of the two investigators to determine the interobserver reliability [8] (Fig. 2).

Equation 1*Dice similarity index (DSI)*:

1 $${\text{DSI}}=\frac{{2 \times \left( {A~\mathop \cap \nolimits^{} B} \right)}}{{A+B}}.$$

To determine the amount of overlap between the researchers, the DSI was implemented as the DSI compares the similarity of two samples. A and B represent the two segmentations, one of each independent investigators.

The outcome of the DSI lies between 0 (low agreement) and 1.0 (high agreement). To measure the distance error, the Euclidean distance between the centroids of the two masks was measured. The Euclidean distance is the straight-line distance between two points in Euclidean space. The Euclidean space in this case was the resolution of the MRI scan and digitized histological section as measured by voxel size. The centroids of the three-dimensional masks need to be created as Euclidean space measures the distance between two points, not two volumes. Assuming a spheroid configuration of the masks, the Euclidean distance between the centroids is an accurate representation of the distance error between the volumes. After this, the distance between the two centroids was measured by plotting Eq. 2.

Equation 2*Euclidean distance*:

2 $${\text{Euclidean}}\;{\text{space}}=\sqrt {{{\left( {C,{\text{FA}}\left( x \right) - C,{\text{Hist}}\left( x \right)} \right)}^2}+~{{\left( {C,{\text{FA}}\left( y \right) - C,{\text{Hist}}\left( y \right)} \right)}^2}+~{{\left( {C,{\text{FA}}\left( z \right) - C,{\text{Hist}}\left( z \right)} \right)}^2}^{~}} .$$

The Euclidean distance or Euclidean metric is the “ordinary” straight-line distance between two points in Euclidean space. With this distance, Euclidean space becomes a metric space. C,FA = centroid of the mask in the FA-maps; C,Hist = centroid of the mask in the histological sections; x;y;z: coordinates of the section planes (axial, sagittal, coronal).

## Results

### Histology

**Fig. 3:**
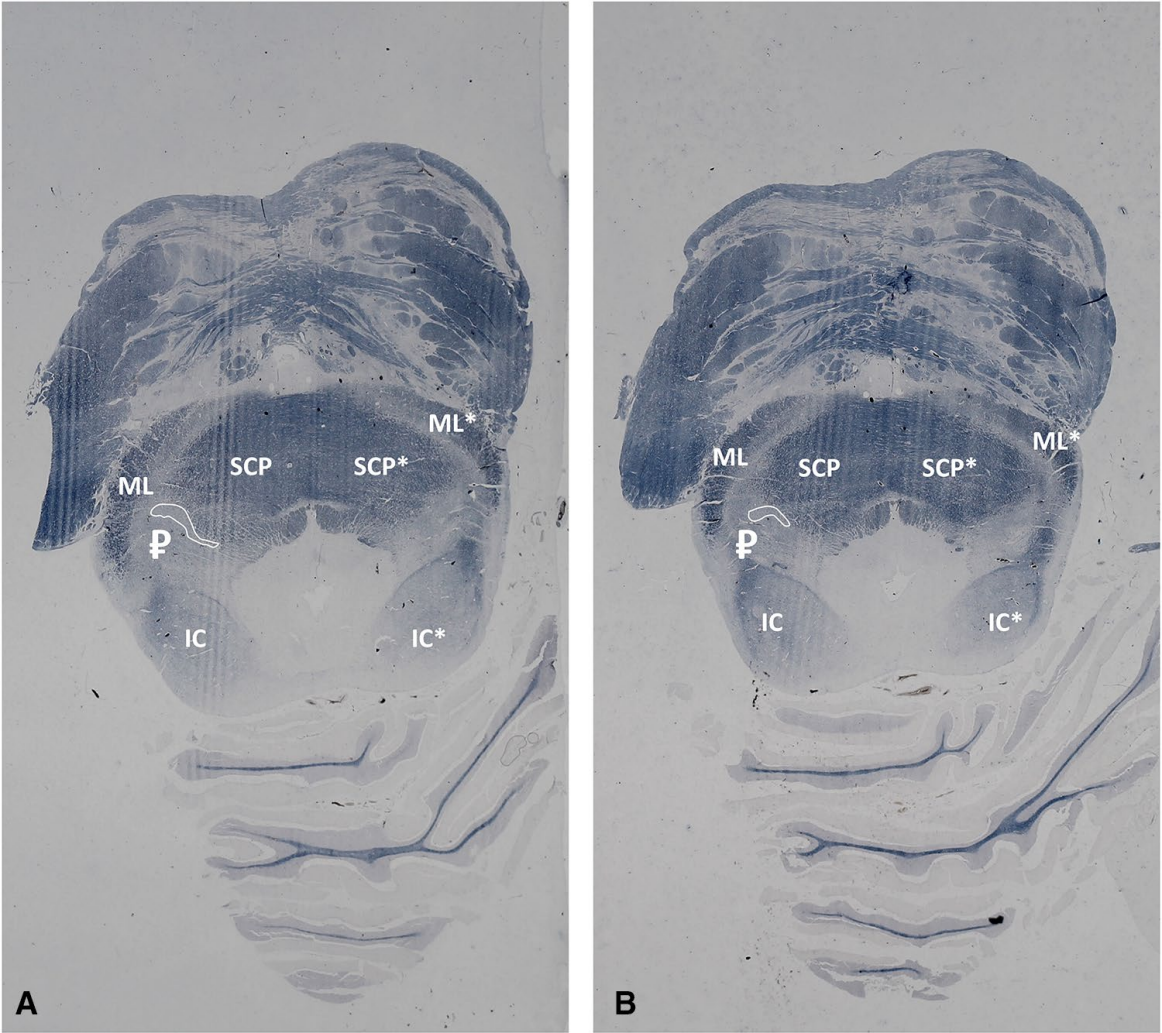
Histological sections of the midbrain after applying the modified Heidenhain-Woelke stain. The modified Heidenhain–Woelke stain was applied to visualize the white matter tracts that make up the PPN region. The unstained areas are therefore gray matter structures. Based on anatomical knowledge, the PPN could be localized in between the ML and the SCP. *IC* Inferior colliculus, *ML* medial lemniscus, *SCP* superior cerebellar peduncle; ₽ pedunculopontine nucleus, *Left-sided neural structures

On the transverse histological sections, at the level of the inferior colliculus the SCP and the ML can be recognized. In between, near the dorsolateral portion of the SCP, unstained tissue appears to be present. This allantoid-shaped gray matter, with its long axis parallel to the neural axis, is recognized as the PPN. The caudal apex runs in a dorsocaudal direction towards the locus coeruleus (Fig. 3a). More caudal transverse sections through the brainstem, still at the level of the inferior colliculus, also show the allantoid-shaped PPN, compressed between the SCP medially and the ML laterally. The caudal apex was observed to diminish in size (Fig. 3b).

### Structural MRI and DTI

**Fig. 4:**
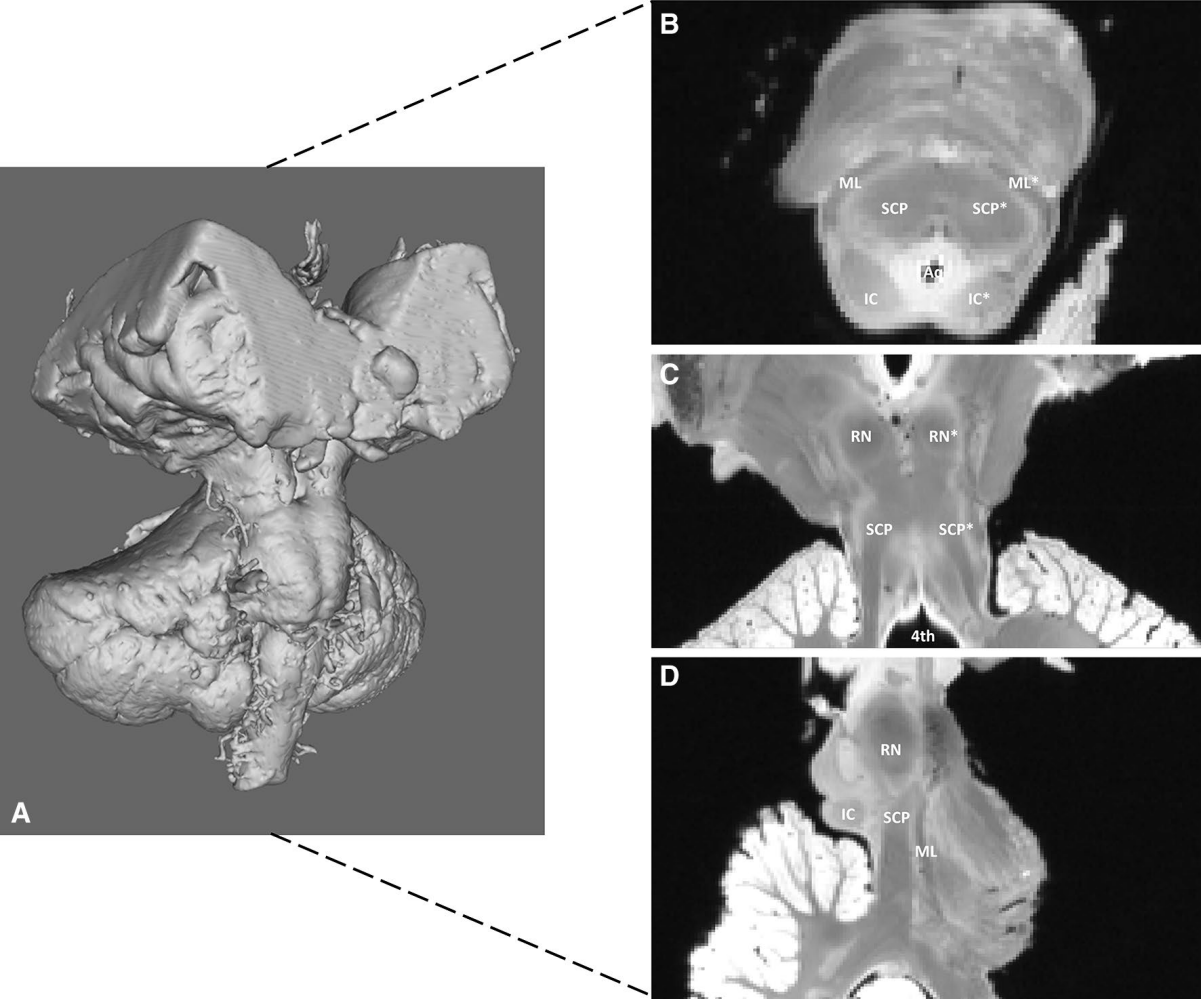
7T, T1-weighted MR scan of post-mortem specimen. **a** Three-dimensional reconstruction of the post-mortem, scanned specimen. **b** Transverse mesencephalic section at the level of the inferior colliculus. The PPN cannot be distinguished from other neural structures. *Aq* Aqueduct, *IC* inferior colliculus, *ML* medial lemniscus, *SCP* superior cerebellar peduncle, *Left-sided neural structures. **c** Coronal section at the level of the decussation of the SCP. The PPN cannot be distinguished from other neural structures. *RN* Red nucleus, *SCP* superior cerebellar peduncle, *4th* fourth ventricle, *Left-sided neural structures. **d** Paramedian sagittal section. The PPN cannot be distinguished from other neural structures. *IC* Inferior colliculus, *ML* medial lemniscus, *SCP* superior cerebellar peduncle, *RN* red nucleus

**Fig. 5:**
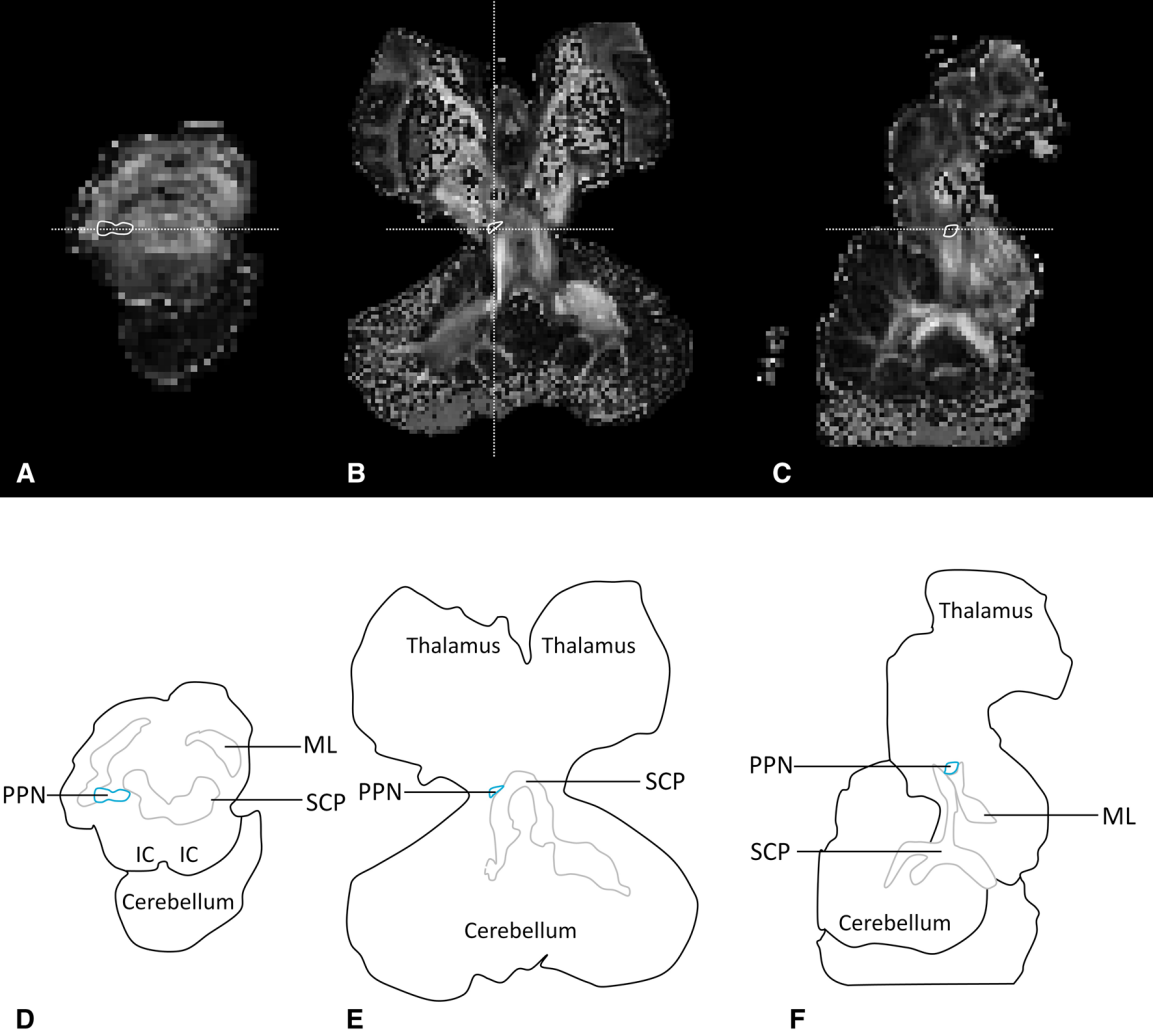
7T, diffusion-weighted MR scan (fractional anisotropy map) of post-mortem specimen. Fractional anisotropy (FA) shows to be low in the region of the PPN, which causes a black spot in both the transverse and sagittal sections, surrounded by the higher FA-values of white matter tracts. **a** Transverse section at the level of the inferior colliculus, at the level of the horizontal white dashed line depicted in **c**. The PPN can be seen as a FA-low region surrounded by higher FA structures, including the superior cerebellar peduncle and the medial lemniscus. **b** Coronal section at the level of the horizontal white dashed line depicted in **a**, at the level of the PPN. The PPN can be seen as a FA-low region surrounded by higher FA structures, including the superior cerebellar peduncle. **c** Paramedian, sagittal section at the level of the vertical white dashed line depicted in **b**. The PPN can be distinguished from its surrounding as a FA-low region. The PPN appears to lie rostroventral to the superior cerebellar peduncle and dorsal to the rostral extend of the medial lemniscus. Both aforementioned white matter tracts have higher FA-values compared to the PPN itself. **d** Schematic overview of transverse section in **a**. *IC* Inferior colliculus, *ML* medial lemniscus, *PPN* pedunculopontine nucleus, *SCP* superior cerebellar peduncle. **e** Schematic overview of coronal section in **b**. *PPN* Pedunculopontine nucleus, *SCP* superior cerebellar peduncle. **f** Schematic overview of sagittal section in **c**. *ML* Medial lemniscus, *PPN* pedunculopontine nucleus, *SCP* superior cerebellar peduncle

In all three planes of the structural 7T MRI, at the presumed position of the PPN, hardly any contrast to surrounding tissue appears present (Fig. 4), which complicates recognition of the PPN using only the structural MR images. Using the FA-maps, acquired from BEDPOSTX estimations of each voxel, an isotropic area can be distinguished at the level of the inferior colliculus. Surrounded by anisotropic fields, the isotropic area can be easily observed (Fig. 5). Using the sagittal planes, the anisotropic fields can be recognized as the SCP (Left SCP FA-value = 0.46; Right SCP FA-value = 0.42) and the ML (Left ML FA-value = 0.64; Right ML FA-value = 0.68). The isotropic area is therefore recognized as the PPN (Left PPN FA-value = 0.19; Right PPN FA-value = 0.18).

### Dice similarity index and Euclidean distance

First, the histological segmentations were compared. A DSI of 1.0 was found with regard to the two independently performed PPN segmentations in the histological sections. This indicates that full agreement between the observers was achieved, demonstrating the superiority of histology. The segmentations of the PPN in the DTI FA-maps were subsequently compared. This showed a DSI of 0.82, which shows that the PPN can also be recognized on DTI FA-maps with high certainty. To compare the overlap between the four segmentations in the two modalities, the segmentations in the histological images and DTI FA-map were compared in two fashions. First, the DSI of the overlap-mask of the DTI FA-maps and the histological sections showed to be 0.32. Second, the DSI of the concatenation-masks of the DTI FA-maps and the histological sections showed to be 0.94 (Fig. 2). Voxel dimensions [x;y;z] of the centroids of the overlap mask in the FA-map were [210.0421; 281.9907; 193.4486] Voxel dimensions [x;y;z] of the centroids of the overlap-masks in the histological sections showed to be [206.9231; 280.7692; 189.5020] Euclidean distance between the centroids of the overlap-masks of the FA-map and histological slices showed to consist of 5.1764 voxels (each voxel being 0.25 mm isotropic in the structural space) or 1.29 mm. Voxel dimensions [x;y;z] of the centroids of the concatenation-mask in the FA-map were [210.7323; 280.6826; 193.9847]. Voxel dimensions [x;y;z] of the centroids of the concatenation-masks in the histological sections showed to be [206.3020; 280.4816; 189.4265]. Euclidean distance between the centroids of the concatenation-masks of the FA-map and histological slices showed to consist of 6.3596 voxels (each voxel being 0.25 mm isotropic in the structural space) or 1.59 mm.

## Discussion

This study shows that 7T DTI FA-maps can be used to identify the regional anatomy surrounding the PPN and its location with considerable accuracy (at 1.0 mm isotropic resolution). Furthermore, it was observed that a correlation exists between a local minimum of the fractional anistropy at the level of the inferior colliculus, at the top of the SCP and the localization of the PPN. The results show important similarities to the findings by Alho et al., thereby providing further proof that the FA could be of added clinical value in targeting of the PPN. Furthermore, histological visualization of white matter tracts that surround the PPN (i.e., SCP and ML) shows to be an accurate method to visualize the PPN in an indirect fashion, due to the white matter/gray matter contrast of these structures. Recently, the study of Alho et al. shows for the first time, the neuroanatomical landmarks of the PPN as observed in particular MR sequences with the corresponding histological data of the same brain [3]. In their methodology, a Nissl staining was applied to visualize the PPN. However, it is well known that NADPH diaphorase and choline acetyltransferase staining still constitute the best and definite way of identifying the extent of the PPN and differentiating it from other surrounding neural tissues. In our study, a myelin staining protocol was implemented as it was the aim to study the anatomical, white matter borders of the PPN and thereby, the PPN itself indirectly. The lack of staining in between large white matter tracts was identified as the PPN. Our study is the first to report that this void in the white matter tracts at the level of the PPN shows to be a fair method to identify the PPN. Furthermore, the results from Alho et al. with regard to the diffusion parameters of the PPN and its surroundings could be reproduced at MR imaging methods using much higher magnetic fields. In a recent study, a multi-modal imaging approach using 7T MRI enables accurate identification of the PPN by combining DTI and susceptibility weighted imaging (SWI) in non-human primate brainstems. However, to the authors’ knowledge, no other studies have used SWI solely to identify the PPN in the brainstem [30]. This is relevant as subcortical gray matter is known to have paramagnetic, diamagnetic and/or ferromagnetic properties, which all interact with the local magnetic field of the MRI. Although this interaction can alter the DTI signal, the PPN is not known to have the aforementioned properties, which makes DTI more reliable to use as an indirect visualization method of localizing the PPN. Taking the present study into account, the authors would recommend clinical use of a 7T scanning protocol to delineate the PPN as delineation at this field strength is thought to be more accurate.

The presented local minimum in FA-values can be explained by the relative high number of neuronal nuclei within the PPN compared to surrounding tissue comprised of axons [2]. As DTI and DW-MRI measure diffusion of water molecules without fundamental relation to anatomical structures, the conclusion that a local reduction of fractional anisotropy overlaps with gray matter structures must be validated with histological evaluation. The DSI of the overlap-masks and concatenation-masks between the DTI FA-maps and the histological sections show considerable differences in overlap. The concatenation-maps show the greatest overlap, suggesting that the differences in DTI FA-maps between both investigators are not necessarily false-positive areas of the masks. Another explanation of the differences in DSI of both types of masks can be found in the fact that the center of the PPN is less anisotropic compared to its borders. This indicates that manual segmentation can be challenging. The relatively low DSI between overlap-masks of the DTI FA-images and the histological sections can be explained by the slightly diminished quality of the more cranial sections in the used dataset which confounds the linear registration of both datasets in this area. Furthermore, DSI overlap between the two modalities (MRI vs. histology) might be influenced due to deformation after histological processing. However, this possible limitation was aimed to be averted by use of multiple linear transformation, which resulted in a great amount of overlap between the histological slices and the MR images (Fig. 1). Nevertheless, it must be underlined that although the overlap between DTI FA-images and histological sections seems relatively poor, the PPN cannot even be distinguished at 7T structural MR images. A recent review advocates the use of T2-weighted and proton density sequences for direct visualization of landmarks in the region of the PPN, whereas other investigators advise to use of major white matter trajectories or the borders of the inferior colliculus as anatomical landmarks of the PPN [11]. According to this study, indirect visualization of the PPN using its DTI characteristics and those of its white matter borders provides a fair visualization method that could be feasible in the clinical setting in the future. It should be noted that the presented dataset has not been acquired in a clinical setting. The 7T post-mortem scan had a duration of scanning of approximately 32 h and a small knee coil was used for data acquisition. Although 7T MR imaging is already implemented in clinical radiology [16], the duration of scanning is not feasible in clinical settings. Improvements of the current targeting method can be found in many fields of research. For example, successful implementation of high-quality DWI MRI and DTI is thought to be critical for implementation of DTI based PPN targeting. High-resolution DWI MRI and DTI are known to suffer from inherent low signal-to-noise ratio, further decreased by a reduction in T2 and T2*. This T2* reduction combined with a high B0 is the source of artifacts, blurring and distortions, degrading the image quality. Fortunately, promising methods have been developed for 7T DW-MRI and DTI leading to promising quality image data at high-resolutions [29]. Studies also reported a reduction of the diffusivity values in post-mortem white matter. In 2007, D’Arceuil reported a significant decrease of regional ADC and FA-values in gray and white matter over time after death. However, at a post-mortem interval of 14 days, still a detectable diffusion anisotropy in the major white matter was present [6]. Furthermore, compared to in vivo parameters, all diffusivity measures dropped after death and further declined following fixation, with FA being the only exception [24]. To overcome this possible shortcoming, a relatively short postmortem interval was employed in this study. The period after fixation is not regarded as a possible limitation as the diffusivity measures have been suggested to remain stable up to 3 years after fixation [9]. Another applied methodology to overcome these challenges was found in the scanning protocol. The implemented DW-SSFP sequence is reported to achieve strong diffusion weighting without unacceptable T2 signal loss [18].

One of the strengths of this paper lies in the applied methodology, using both high-resolution MR imaging and histological examination with two independent observers. The major limitation of this studies’ methodology is made up by the limited sample size (*n* = 1) and therefore, extrapolation of these findings to the general population might be challenging. Another particular strength of the presented visualization technique is that it can be used to visualize the PPN itself, not its region. However, the authors believe this study can be an initial step towards in vivo PPN targeting. Furthermore, the current FA-maps as a method used to identify the PPN might not be the only method to create contrast between the PPN and surrounding tissue. Additional research is thought to be able to develop new methods, as well as refine the one described in this study and previous work in PPN targeting.

## Conclusion

This study supports previous findings that the PPN can be identified using DTI FA-maps. For possible clinical application in DBS localization, in vivo validation of the findings of our study is needed.
